# Phase I study to assess safety, biodistribution and radiation dosimetry for ^89^Zr-girentuximab in patients with renal cell carcinoma

**DOI:** 10.1007/s00259-021-05271-w

**Published:** 2021-03-02

**Authors:** Robin I. J. Merkx, Daphne Lobeek, Mark Konijnenberg, Luis David Jiménez-Franco, Andreas Kluge, Egbert Oosterwijk, Peter F.A. Mulders, Mark Rijpkema

**Affiliations:** 1 Department of Medical Imaging: Nuclear Medicine, Radboudumc, Geert Grooteplein Zuid 10, 6525 GA Nijmegen, The Netherlands; 2 Department of Urology, Radboudumc, Nijmegen, The Netherlands; 3 ABX-CRO advanced pharmaceutical services, Dresden, Germany

**Keywords:** Organ-based dosimetry, RCC, Girentuximab, Zirconium-89

## Abstract

**Purpose:**

In this phase I study, we evaluated the safety, biodistribution and dosimetry of [^89^Zr]Zr-DFO-girentuximab (^89^Zr-girentuximab) PET/CT imaging in patients with suspicion of clear cell renal cell carcinoma (ccRCC).

**Methods:**

Ten eligible patients received an intravenous administration of 37 MBq (± 10%) of ^89^Zr-girentuximab at mass doses of 5 mg or 10 mg. Safety was evaluated according to the NCI CTCAE (version 4.03). Biodistribution and normal organ dosimetry was performed based on PET/CT images acquired at 0.5, 4, 24, 72 and 168 h post-administration. Additionally, tumour dosimetry was performed in patients with confirmed ccRCC and visible tumour uptake on PET/CT imaging.

**Results:**

^89^Zr-girentuximab was administered in ten patients as per protocol. No treatment-related adverse events ≥ grade 3 were reported. ^89^Zr-girentuximab imaging allowed successful differentiation between ccRCC and non-ccRCC lesions in all patients, as confirmed with histological data. Dosimetry analysis using OLINDA/EXM 2.1 showed that the organs receiving the highest doses (mean ± SD) were the liver (1.86 ± 0.40 mGy/MBq), the kidneys (1.50 ± 0.22 mGy/MBq) and the heart wall (1.45 ± 0.19 mGy/MBq), with a mean whole body effective dose of 0.57 ± 0.08 mSv/MBq. Tumour dosimetry was performed in the 6 patients with histologically confirmed ccRCC resulting in a median tumour-absorbed dose of 4.03 mGy/MBq (range 1.90–11.6 mGy/MBq).

**Conclusions:**

This study demonstrates that ^89^Zr-girentuximab is safe and well tolerated for the administered activities and mass doses and allows quantitative assessment of ^89^Zr-girentuximab PET/CT imaging in patients with suspicion of ccRCC.

**Trial registration:**

NCT03556046—14th of June, 2018

**Supplementary Information:**

The online version contains supplementary material available at 10.1007/s00259-021-05271-w.

## Introduction

Renal cell carcinoma (RCC) accounts for 5% and 3% of all cancers worldwide for men and women, respectively [1]. Renal tumours are diverse and their clinical behaviour is highly dependent on the histological subtype [2]. Of these, clear cell RCC (ccRCC) is the most common subtype and accounts for the majority of kidney cancer-related deaths [3]. The accuracy and generalizability to distinguish ccRCC from non-ccRCC with multiphasic enhanced imaging such as magnetic resonance imaging (MRI) or computed tomography (CT) is debatable [4]. In order to inform patient risk stratification and prevent overtreatment of benign/low-grade lesions, renal tumour biopsies (RTB) are performed. While RTB has demonstrated to be reliable in determining the presence of malignancy, the low negative predictive value remains an issue [5]. Additionally, tumour biopsies are often restricted to a single site and thus fail to provide information on the extent of disease. Therefore, a method to non-invasively identify ccRCC in both primary and metastatic disease provides valuable information. Approximately 95% of all ccRCC have an overexpression of the antigen carbonic anhydrase IX (CAIX) on the surface of tumour cells due to a mutation of the von Hippel-Lindau (VHL) protein [6, 7]. The high expression of CAIX in ccRCC in combination with the very limited expression in normal tissue and non-ccRCC lesions endorses CAIX as an excellent marker for the distinction between ccRCC and non-ccRCC [8]. CAIX can be effectively targeted by the chimeric monoclonal antibody girentuximab [9]. Multiple studies describe the high accuracy and clinical benefit of PET/CT imaging using radiolabeled girentuximab (i.e. [^124^I]I-girentuximab (^124^I-girentuximab) and [^89^Zr]Zr-DFO-girentuximab (^89^Zr-girentuximab)) compared to CT imaging [10–12]. Furthermore, animal studies demonstrate that ^89^Zr-girentuximab provides images with better contrast and spatial resolution compared with ^124^I-girentuximab due to an increased tumour retention of ^89^Zr [13, 14].

Due to this specific targeting, girentuximab is also considered an excellent carrier for radioimmunotherapy (RIT) in tumours with high CAIX expression including metastasized ccRCC. In studies of single-agent CAIX-targeted RIT with [^177^Lu]Lu-DOTA-girentuximab (^177^Lu-girentuximab), a therapeutic response has been demonstrated in patients with pre-treated RCC, with myelotoxicity identified as the most common clinically relevant adverse finding [15, 16]. The use of personalized dosimetry of tumours and organs at risk would allow for a better prediction of the achievable therapeutic value of CAIX-targeted RIT [17]. This could improve patient selection for this treatment and potentially prevent unnecessary toxicity. In order to estimate the biodistribution and radiation dose of girentuximab-bound therapeutic radionuclides, the positron emitter ^89^Zr could function as an analogue when labeled to the same antibody in a theranostic approach [18]. However, clinical data on the safety, biodistribution and dosimetry of ^89^Zr-girentuximab are currently lacking. Therefore, this study aims to assess the safety, biodistribution and dosimetry of ^89^Zr-girentuximab in ten patients suspicious for ccRCC.

## Materials and methods

### Study design and patients

This single-centre, prospective phase I study was approved by the Regional Internal Review Board (CMO Arnhem Nijmegen; ClinicalTrials.gov identifier NCT03556046). The study had a primary objective of safety and tolerability and secondary objectives of determining the whole body radiation dosimetry of ^89^Zr-girentuximab in patients with suspected ccRCC. The study was open to patients who provided written informed consent and met the following eligibility criteria: patients with a clinical suspicion of primary ccRCC or with established diagnosis of ccRCC suspected for recurrence or metastasis; at least 50 years old; and life expectancy of at least 6 months. Exclusion criteria included uncontrolled hyperthyroidism; exposure to experimental diagnostic/therapeutic drug or any radiopharmaceutical within 30 days prior to administration of ^89^Zr-girentuximab; or established renal cell carcinomas of a histological subtype other than ccRCC. A histological specimen was obtained by either biopsy or surgery to formally characterize the tumour.

### Synthesis of ^89^Zr-girentuximab

Conjugation of N-succinyldesferrioxamine-B-tetrafluorphenol (N-SucDf-TFP/DFO) (VU Medical Centre, Amsterdam, The Netherlands) to girentuximab (girentuximab was provided Telix Pharmaceuticals, Melbourne, Australia) was performed as described previously [19]. In short, 3 days before injection, 2 mg of DFO-girentuximab was radiolabeled with 90 MBq of zirconium-89 (Perkin Elmer, The Netherlands). The radiolabeling process was performed at a pH of 7.2. To achieve the desired pH value, oxalic acid, sodium carbonate and HEPES buffer (adjusted to pH 7.3 by use of sodium hydroxide solution) were added. The radiolabeling was carried out during 60 min at room temperature. Next, unbound ^89^Zr was complexed by the chelator ethylenediaminetetraacetic acid (EDTA) by incubation for 15 min at room temperature. Then the product was purified using gel filtration on a disposable PD10 column. The protein dose was increased to either 5 mg or 10 mg by adding unlabelled girentuximab. Radiochemical purity was determined by high-performance liquid chromatography (HPLC) and exceeded 90%. The end product was diluted to a total volume of 10 ml with NaCl 0.9%.

### ^89^Zr-girentuximab administration

A single injection of 37 MBq (± 10%) ^89^Zr-girentuximab was administered intravenously over approximately 2 min. Post-administration (p.a.), the syringe was flushed once using 10 mL NaCl 0.9%. The intravenous line and syringe were measured for residual activity. Patients were monitored for 30 min after injection. Adverse events were graded according to Common Terminology Criteria for Adverse Events (CTCAE version 4.03). Patients were randomized in a ratio of 1:1 to receive the ^89^Zr-girentuximab in a mass dose of 5 mg or 10 mg.

### Safety assessment

In order to comprehensively characterize safety and tolerability of ^89^Zr-girentuximab, a set of standard parameters, including physical exam, vital signs, haematology, serum chemistry, urinalysis and 12-lead electrocardiograms (ECGs) were assessed at predefined time intervals (screening, pre-administration, 24 h p.a. and 8 days (± 1 day) p.a.).

### ^89^Zr-girentuximab PET/CT imaging

For the purpose of internal radiation dosimetry, whole body (i.e. base skull to the upper thighs) PET/CT imaging was performed at 5 time points: 0.5, 4, 24, 72 and 168 h p.a. The PET data were acquired on a Siemens Biograph mCT 4/40 scanner (Siemens, Munich, Germany) using list mode and time of flight (ToF) for 6–8 bed positions with 5 min per bed position. The PET images were reconstructed using a 3D ordered subset expectation maximization (OSEM) algorithm with a spatially varying point-spread function and ToF. Image reconstruction was performed with 3 iterations, 21 subsets and a matrix size from 128 × 128 (whole body imaging) to 300 × 300 pixels (tumour imaging). At 72 h and 168 h p.a., additional PET data of the targeted lesion was acquired using 1 bed position for 20 min in list mode using the same settings. PET imaging was preceded by a low-dose CT (ldCT) scan for attenuation correction and anatomical reference. Additionally, a diagnostic contrast-enhanced CT (ceCT) image was acquired within 30 days before treatment for tumour volume measurements.

### Dosimetry

Patient imaging data were loaded to QDOSE dosimetry software suite (ABX-CRO Advanced Pharmaceutical Services Forschungsgesellschaft, Dresden, Germany) for processing and analysis. All ldCT images were coregistered using automatic rigid coregistration followed by deformable coregistration. Manual coregistration between PET images and their corresponding ldCT images was performed when necessary.

For safety dosimetry calculation, the following source organs were included: kidneys, liver, spleen, heart content, red marrow and remainder body. The time activity curve (TAC) for these organs was retrieved from each PET/CT image by determining volumes of interest (VOIs) fully containing each organ (except for the red marrow and the remainder body). The activity in the red marrow was estimated by retrieving the activity in the lumbar vertebrae L1–L5, which contain approximately 12.3% of the total red marrow, and extrapolating it to the total red marrow [20]. Source organ and tumour segmentation was performed by manually drawing volumes of interests (VOIs) on either the PET image or its corresponding CT image, copied to other time points, checked and manually adjusted if necessary, in order to retrieve the TACs for all organs and tumour tissue. The TAC of the tracer in the remainder body was determined by a VOI that contained the complete PET field of view.

In order to assess the TACs in the kidneys in patients with renal tumours, the activity in the contralateral healthy kidney was assessed and doubled. In patients with a solitary kidney and a renal tumour, the tumour was excluded from the kidney VOI. The anatomical tumour volumes were segmented on the pre-study diagnostic ceCT for assessment of the tumour masses.

TACs were fitted either to a mono- or bi-exponential function depending on the shape of the curve and the duration of the activity accumulation phase. Subsequently, source organ and tumour lesion TACs were integrated from time 0 min to infinity and divided by the injected activity to obtain time-integrated activity coefficients (TIACs) for all source organs and tumour lesions. The TIAC for the remainder body was calculated using the effective half-life of the radiotracer in the body, followed by subtraction of the TIACs from the other source organs.

The TIACs for the source organs were subsequently entered as an input to OLINDA/EXM 2.1 for dose calculations [21]. Organ masses were not adapted to individual subject organ masses for dose calculations. For the sake of a comparison with studies using OLINDA/EXM 1.1 with other ^89^Zr-labelled monoclonal antibodies (mAbs), dose calculations were also performed using OLINDA/EXM 1.1.

The tumour-absorbed doses were determined using a spherical model incorporated in OLINDA/EXM 2.1 in which the tumour TIACs and specific tumour masses (obtained from the segmented tumour volumes assuming a tumour density of 1.03 g/cm^3^) were used as an input. Additionally, extrapolation of the tumour-absorbed doses to the potential therapeutic beta-emitter ^177^Lu was performed by recalculating the tumour TIACs when replacing the ^89^Zr physical decay for the physical decay of ^177^Lu.

### Statistical analysis

Absorbed and effective dose estimates were individually calculated for each patient. Subsequently, these data were summarized using descriptive statistics and reported using mean, median and standard deviation (SD).

## Results

### Patients

Ten patients (68 ± 8 years; range 55 to 77 years; 8 males) were enrolled in the study. Eight patients had a suspicion of a primary renal tumour and two patients had a suspicion of recurrence or metastasis of known ccRCC (Table 1). All patients were eligible for dosimetric analysis.

**Table 1:** Patient characteristics of 10 patients with RCC suspicion

Patient	Age (y)	Gender (M/F)	Mass dose (mg)	Primary or metastatic lesion	Sites of suspicious lesion(s) and size^a^	Type surgery/biopsy	Days between tissue harvest and PET/CT	Positive PET/CT imaging (Y/N)	Pathology outcome
1	73	M	5	Primary	Right kidney (31 mm)	PN	7	N	No malignancy
2	76	M	5	Primary	Left kidney (61 mm)	PN	21	Y	ccRCC
3	61	M	5	Primary	Left kidney (83 mm)	RN	28	Y	ccRCC
4	71	M	10	Primary	Right kidney (58 mm)	RN	70	Y	ccRCC
5	76	F	10	Primary	Right kidney (42 mm)	RN	63	N	Papillary RCC
6	57	M	10	Metastasis	Left kidney (20 mm), left adrenal gland (23 mm)	N/A	N/A	Y^b^	ccRCC^c^
7	77	M	5	Primary	Right kidney (49 mm)	Biopsy	41	N	Chromophobe RCC
8	63	M	10	Primary	Left kidney (35 mm)	PN	21	Y	ccRCC
9	55	M	10	Primary	Bilateral kidneys (37 and 26 mm)	Biopsy (right)	63	N	Papillary RCC
10	68	F	5	Metastasis	Right adrenal gland (21 mm)	Adrenalectomy	101	Y	ccRCC

^a^Size of lesion in the largest diameter (millimetre). ^b^ PET/CT imaging showed accumulation of ^89^Zr-girentuximab in a mediastinal lesion (10 mm) which was not suspect beforehand. ^c^ Based on historical pathology results of primary renal tumour in the right kidney. *N/A*, not applicable; *PN*, partial nephrectomy; *RN*, radical nephrectomy

### Safety evaluation

The mean administered activity was 36.7 ± 1.1 MBq (range 34.2 to 38.34 MBq) of ^89^Zr-girentuximab. A total of 7 adverse events (AE) were reported in 4 patients. One SAE, post-operative bleeding (grade 3), was reported but not considered to be related to administration of the investigational product. No adjustment of dosing or administration occurred due to AEs. All remaining AEs were graded as mild and ranged from common cold to nausea (Supplementary Table 1). No significant changes in vital signs, laboratory results or ECGs were observed (data on file).

### PET/CT imaging

All PET/CT imaging was evaluated by a single, experienced nuclear medicine physician. The criteria for tumour uptake were binary based on visual assessment. In all patients, ^89^Zr-girentuximab PET/CT imaging allowed successful differentiation between ccRCC and non-ccRCC lesions which was confirmed with the reference histology specimens.

**Fig. 1:**
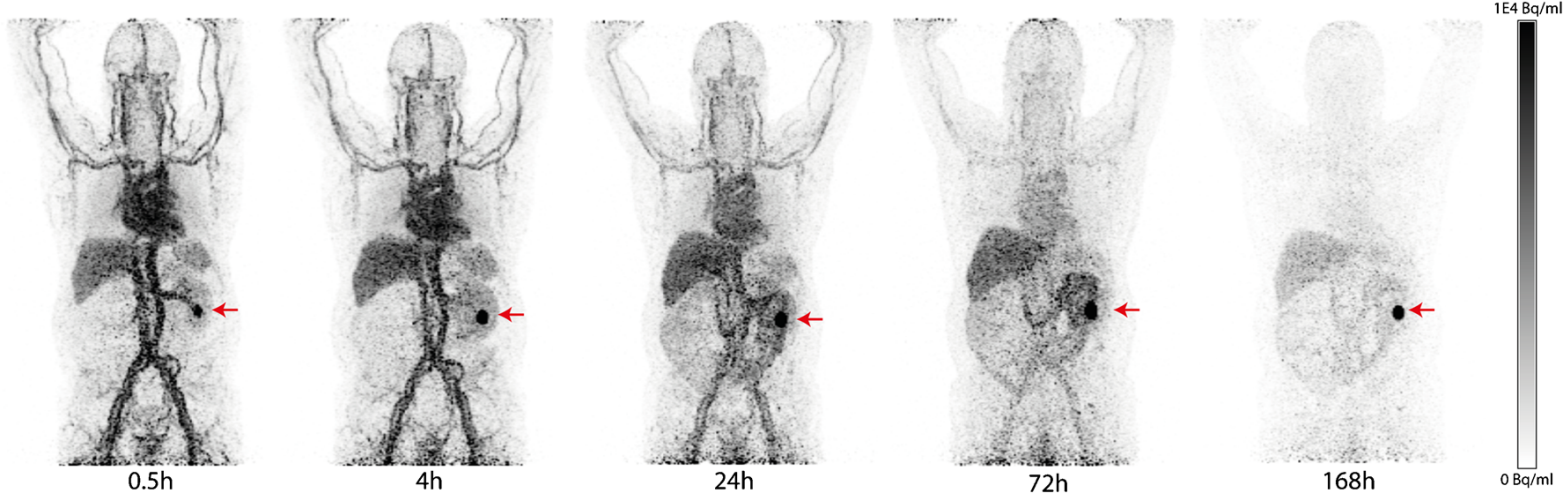
MIP of patient #8 (male 63 years. Mass dose of 10 mg girentuximab) demonstrating a ccRCC tumour in left kidney (red arrow) at 0.5 h p.a. with an increased tumour to background ratio over time. MIP, maximum intensity projection

In six patients (patients 2, 3, 4, 6, 8 and 10), ^89^Zr-girentuximab uptake was observed in the suspicious lesion(s). In most patients, tumour lesions became visible on PET imaging at 24 h p.a. after which subjective visualization improved. No additional lesions were detected between ~ 72 and ~ 168 h p.a. in any patient. In patient 8, the tumour lesion was already visible at 0.5 h p.a. (Fig. 1).

**Fig. 2:**
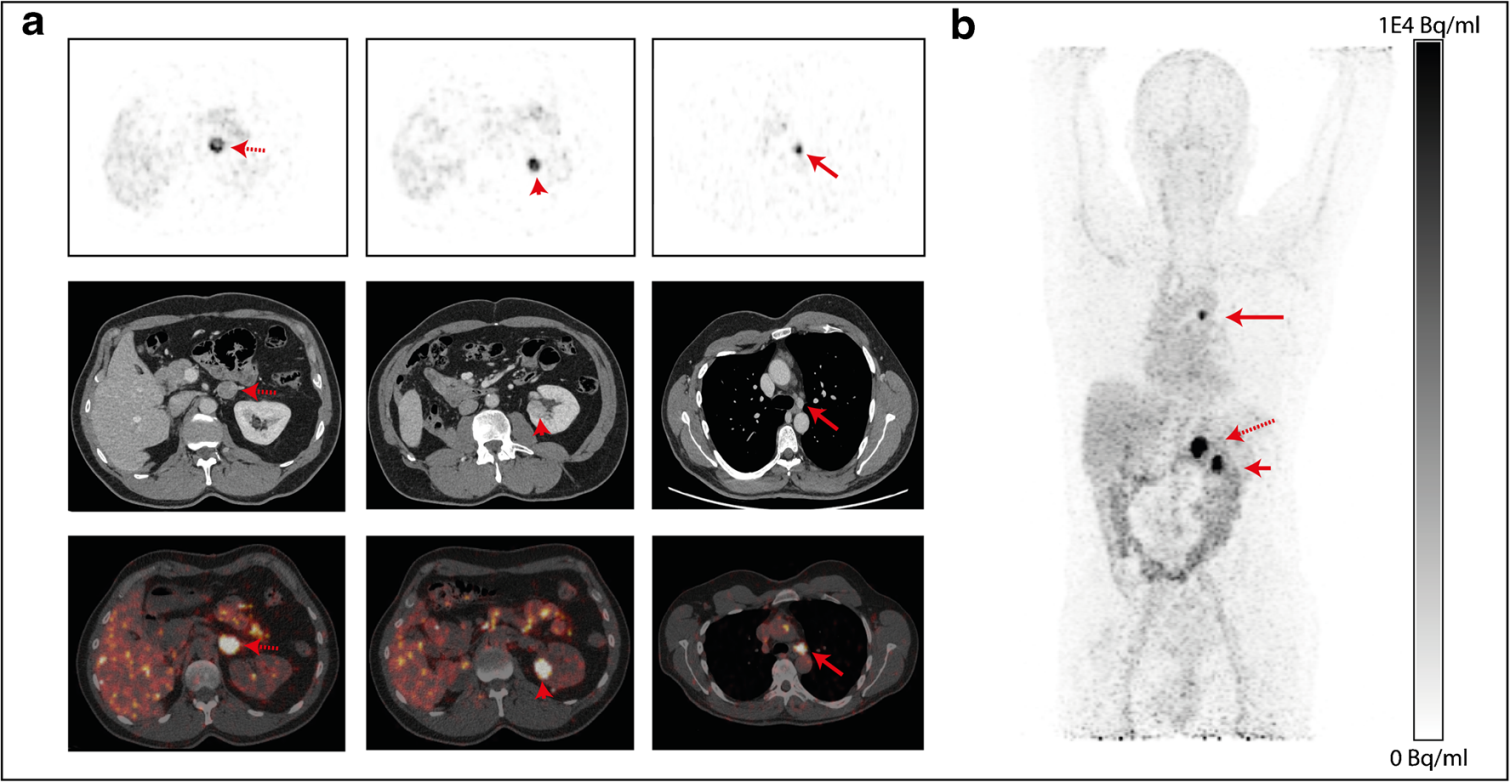
**a** PET imaging (168 h p.a.; upper row). ceCT (middle row) and fused imaging (lower row) of patient #6 (male 57 years. Mass dose 10 mg girentuximab) showing ^89^Zr-girentuximab uptake in the adrenal gland (red dotted arrow). Left kidney (red arrowhead) and mediastinal lymph node (red arrow) from left to right. **b** The MIP of patient #6 at 168 h p.a.; p.a., post-administration; MIP, maximum intensity projection

In patient 6, who had a history of ccRCC, three ^89^Zr-girentuximab-avid lesions were detected in the left kidney, left adrenal gland and mediastinal lymph node, respectively (Fig. 2). Interestingly, of these lesions, only two (left kidney and left adrenal gland) were diagnosed on CT imaging. Even though no histological specimen of the metastases was obtained, follow-up CT imaging demonstrated growth of all three lesions, strongly suggesting metastases. In the remaining patients, all lesions with ^89^Zr-girentuximab uptake corresponded to the lesions that were deemed suspicious based on ceCT imaging.

No tumour uptake of ^89^Zr-girentuximab was seen in 4 patients for whom tumour lesions were histologically confirmed as non-ccRCC. In patient 1, the resected specimen showed residual cyst without malignancy; patients 5 and 9 both had confirmed papillary RCC, and patient 7 had confirmed chromophobe RCC.

### Radiation dosimetry

The mean absorbed doses when using OLINDA/EXM 2.1 are given in Table 2 (individual dose values calculated with OLINDA/EXM 2.1 and OLINDA/EXM 1.1 can be found in Supplementary Tables 2 and 3, respectively). Highest absorbed doses were seen in the liver (1.86 mGy/MBq), kidneys (1.50 mGy/MBq), heart wall (1.45 mGy/MBq), adrenals (1.07 mGy/MBq) and spleen (1.03 mGy/MBq). The mean whole body effective dose following administration of ^89^Zr-girentuximab was 0.57 ± 0.08 mSv/MBq (range 0.47–0.71 mSv/MBq).

**Table 2:** Absorbed dose to individual organs for ^89^Zr-girentuximab calculated using OLINDA/EXM 2.1

Target organ	Absorbed dose estimates mGy/MBq							
	5 mg (*n* = 5)			10 mg (*n* = 5)				
	Mean	SD	Min	Max	Mean	SD	Min	Max
Adrenals	1.11	0.16	0.85	1.27	1.04	0.10	0.88	1.15
Brain	0.33	0.04	0.30	0.40	0.34	0.05	0.31	0.43
Breasts^a^	0.46	–	–	–	0.47	–	–	–
Oesophagus	0.67	0.08	0.60	0.80	0.66	0.08	0.60	0.80
Eyes	0.34	0.04	0.30	0.41	0.34	0.05	0.31	0.43
Gallbladder wall	1.11	0.20	0.84	1.33	0.96	0.12	0.80	1.13
Left colon	0.62	0.10	0.52	0.78	0.62	0.11	0.55	0.81
Small intestines	0.58	0.05	0.51	0.64	0.57	0.04	0.53	0.65
Stomach wall	0.67	0.06	0.60	0.75	0.66	0.05	0.61	0.75
Right colon	0.66	0.06	0.55	0.71	0.63	0.05	0.57	0.71
Rectum	0.51	0.06	0.45	0.59	0.51	0.07	0.47	0.63
Heart wall	1.40	0.22	1.16	1.66	1.50	0.20	1.30	1.82
Kidneys	1.47	0.32	1.25	2.03	1.53	0.12	1.41	1.68
Liver	2.07	0.45	1.34	2.50	1.65	0.28	1.21	1.98
Lungs	0.59	0.07	0.52	0.70	0.58	0.07	0.53	0.71
Ovaries^a^	0.61	–	–	–	0.65	–		
Pancreas	0.78	0.12	0.64	0.96	0.74	0.10	0.66	0.90
Prostate	0.46	0.03	0.43	0.49	0.46	0.02	0.45	0.48
Salivary glands	0.39	0.03	0.36	0.44	0.40	0.04	0.37	0.47
Red marrow	0.79	0.16	0.63	1.01	0.76	0.09	0.67	0.92
Osteogenic cells	0.63	0.10	0.54	0.77	0.62	0.06	0.58	0.72
Spleen	1.05	0.31	0.60	1.38	1.01	0.28	0.66	1.35
Testes	0.34	0.02	0.33	0.37	0.35	0.01	0.34	0.36
Thymus	0.63	0.06	0.56	0.71	0.65	0.06	0.61	0.76
Thyroid	0.43	0.03	0.40	0.48	0.44	0.04	0.41	0.51
Urinary bladder wall	0.44	0.02	0.42	0.48	0.45	0.01	0.44	0.47
Uterus^a^	0.59	–	–	–	0.62	–	–	–
Effective dose in total body (mSv/MBq)	0.58	0.10	0.47	0.71	0.56	0.08	0.49	0.70

^a^Both cohorts included one woman; hence, no SD is reported. *SD*, standard deviation

### Tumour dosimetry

Tumour dosimetry was performed in 6 patients with a confirmed ccRCC and visible uptake in the tumour region (Table 3). In all 6 patients, the biological accumulation of ^89^Zr-girentuximab was still ongoing at the last time point (data on file). Patient 10 had a nephrectomy prior to inclusion in this study and therefore dosimetry was only performed on a metastasis in the right adrenal gland. The absorbed dose for ^89^Zr-girentuximab in the tumour lesions ranged between 1.90 and 11.6 mGy/MBq with a mean of 4.93 mGy/MBq. The extrapolation to ^177^Lu resulted in tumour-absorbed doses between 2.77 and 23.6 mGy/MBq (Table 3).

**Table 3:** Tumour-absorbed dose for ^89^Zr-girentuximab

Patient	Tumour	Nuclear grade	Pathological dedifferentiation (Y/N)	Estimated tumour mass (g)	Absorbed dose ^89^Zr (mGy/MBq)	Extrapolated absorbed dose ^177^Lu (mGy/MBq)
2	Primary—left kidney	WHO 2	N	81.4	2.10	3.40
3	Primary—left kidney	WHO 4	Y. 40% sarcomatoid	253.4	1.90	2.77
4	Primary—right kidney	WHO 3	N	114.9	3.52	6.73
6	Primary—left kidney	N/A	N/A	6.0	4.54	11.44
8	Primary—left kidney	WHO 2	N	16.6	11.6	23.60
10	Metastasis—right adrenal gland	N/A	N/A	6.8	5.88	14.46
			Mean	77.3	4.93	10.40
			SD	77.4	3.29	7.22
			Median	55.4	4.03	9.09

*N/A*, not applicable

## Discussion

In this phase I study, we demonstrated that intravenously administered ^89^Zr-girentuximab is safe and facilitates feasible dosimetric analyses in patients with primary and advanced RCC. Additionally, we explored the extrapolation of the tumour doses to a therapeutic radiopharmaceutical (^177^Lu-girentuximab) using ^89^Zr-girentuximab as a surrogate.

The mean effective dose of ^89^Zr-girentuximab (0.57 mSv/MBq) is in range with the reported mean effective doses of other ^89^Zr-labelled mAbs (0.36–0.66 mSv/MBq), which have been reported for ^89^Zr-HuJ591, ^89^Zr-trastuzumab, ^89^Zr-pertuzumab and ^89^Zr-cmAb U36 [22–25]. Additionally, the organ-absorbed doses indicate a similar biodistribution to other ^89^Zr-based mAbs with the liver, kidneys and heart wall receiving the highest doses. Although a quantitative comparison between doses of 5 and 10 mg was not included in the objectives of this study, our data suggest a slight increase of hepatic uptake in the 5 mg group. The mechanism behind this is not fully understood, but could partly be explained by the blocking of non-specific liver binding at higher antibody doses, which is in line with previous preclinical observations [26, 27]. Moreover, a recent clinical study with radiolabeled girentuximab demonstrated that a protein dose of 10 mg granted the highest tumour to normal ratio [28]. Furthermore, we demonstrated that for an administered activity of approximately 37 MBq, image time points ranging from 3 to 7 days p.a. are appropriate for visual assessment of ccRCC lesions. While imaging at day 7 could offer an improved tumour to background ratio, no additional lesions were detected between day 3 and 7 in these patients. Similar findings concerning optimal imaging time have been reported in earlier studies with radiolabeled girentuximab as well as other ^89^Zr-mAb tracers [22, 25, 29].

The relatively slow uptake and clearance of antibodies requires labeling with radioisotopes that also have a long physical half-life (i.e. ^89^Zr (t_1/2_ = 78.4 h) or ^124^I (t_1/2_ = 100.2 h)) to achieve optimal imaging. As a result, the radiation burden on the patient when using antibody-based tracers is relatively high in comparison with more rapidly clearing PET tracers such as [^18^F]FDG [30]. This could be considered a limiting factor in the development of antibody-based tracers and therefore it is paramount to reduce the radiation dose where feasible. This study indicates that the administration of ~ 37 MBq of ^89^Zr-girentuximab is sufficient to distinguish ccRCC from non-ccRCC lesions. Since the radiation dose is proportional to the administered activity, optimization of the injected radioactivity (i.e. administration of the minimum activity that guarantees sufficient image diagnostic performance) substantially decreases the overall radiation burden on the patient. Furthermore, this study offers valuable safety data on ^89^Zr-girentuximab administration that is necessary to further progress the clinical implementation of this tracer in ccRCC diagnosis (e.g. differentiation of ccRCC, staging of ccRCC or evaluating response to therapy). In order to assess the diagnostic accuracy of ^89^Zr-girentuximab in renal masses, the multi-centre phase III ZIRCON study has been initiated (NCT03849118).

Girentuximab-based tracers may also be used for therapeutic purposes (i.e. RIT) either alone or in combination with other agents. RIT is based on the delivery of a high dose of therapeutic radiation to cancer cells through tumour antigen–specific targeting. Although this approach has been investigated for several decades, clinical implementation of RIT in solid tumours remains a challenge. The dose-limiting normal tissue in radioimmunotherapy using β-emitters is usually bone marrow [31], as seen in studies that have demonstrated the therapeutic potential of multiple RIT cycles of ^177^Lu-girentuximab in metastasized ccRCC. However, grades 3–4 myelotoxicity precluded additional treatment cycles in several patients [15, 16]. In these studies, a predetermined dose of ^177^Lu-girentuximab was administered to all patients. As a future perspective, PET/CT imaging with ^89^Zr-girentuximab might be a feasible analogue for individualized treatment planning for RIT with girentuximab in advanced ccRCC, which will become even more important with the increasing interest in RIT with alpha-emitting radionuclides. A dosimetry-based theranostic approach is particularly attractive as it allows for an estimation of dose delivery to tumours and normal tissue, thus can be used to guide the selection of patients who are most likely to benefit from RIT [17, 32].

## Conclusion

In the present study, ^89^Zr-girentuximab was found to be safe and well tolerated by all patients after intravenous administration. In addition, PET imaging with ^89^Zr-girentuximab allowed successful differentiation between ccRCC and non-ccRCC lesions in all evaluated patients. The mean effective dose of ^89^Zr-girentuximab was 0.57 mSv/MBq.

## Supplementary information

ESM 1 (DOCX 352 kb)

## Data Availability

The datasets generated during and/or analysed during the current study are available from the corresponding author on reasonable request.
