# Supplementary material for: Phase I study to assess safety, biodistribution and radiation dosimetry for 89Zr-girentuximab in patients with renal cell carcinoma
Source: Eur J Nucl Med Mol Imaging. 2021 Mar 2;48(10):3277–85. doi: 10.1007/s00259-021-05271-w (PMC8426244; doi:10.1007/s00259-021-05271-w)
Supplement: Supplementary file 1 — (DOCX 352 kb) [file 259_2021_5271_MOESM1_ESM.docx]

**Supplementary Table 1.**Adverse Events

| **Patient** | **Adverse Event** | **Mass Dose (mg)** | **Severity (CTCAE v4.03 grade)** | **Relationship to IMP** | **Outcome** | **Serious (Y/N)** |
| --- | --- | --- | --- | --- | --- | --- |
| 1 | Urinary tract infection | 5 | Moderate (2) | Unlikely | Recovered | N |
|  | Post-operative bleeding |  | Severe (3) | None | Recovered | Y |
| 4 | Monoclonal gammopathy of unknown significance | 10 | Mild (1) | None | Ongoing | N |
|  | Headache |  | Mild (1) | Unlikely | Recovered | N |
| 5 | Nausea | 10 | Mild (1) | Possibly | Recovered | N |
|  | Ipsilateral flank pain |  | Mild (1) | Unlikely | Recovered | N |
| 10 | Common cold | 5 | Mild (1) | Unlikely | Recovered | N |

CTCAE = Common Terminology Criteria for Adverse Events, IMP = Investigational Medicinal Product

**Supplementary Table 2.**
Individual absorbed dose to organs calculated using OLINDA/EXM 2.1

|  | Absorbed dose (mGy/MBq) | | | | | | | | | | | | |
| --- | --- | --- | --- | --- | --- | --- | --- | --- | --- | --- | --- | --- | --- |
| Patient | 1 | 2 | 3 | 4 | 5 | 6 | 7 | 8 | 9 | 10 | Median | Mean | SD |
| Target organs |  |  |  |  |  |  |  |  |  |  |  |  |  |
| Adrenals | 1.18 | 1.12 | 1.13 | 1.08 | 1.15 | 0.879 | 0.853 | 1.04 | 1.03 | 1.27 | 1.10 | 1.07 | 0.12 |
| Brain | 0.320 | 0.302 | 0.346 | 0.332 | 0.432 | 0.318 | 0.298 | 0.309 | 0.325 | 0.404 | 0.32 | 0.34 | 0.04 |
| Breasts | - | - | - | - | 0.472 |  |  |  |  | 0.455 | 0.46 | 0.46 | 0.01 |
| Oesophagus | 0.665 | 0.626 | 0.657 | 0.642 | 0.797 | 0.603 | 0.601 | 0.621 | 0.630 | 0.797 | 0.64 | 0.66 | 0.07 |
| Eyes | 0.312 | 0.303 | 0.347 | 0.333 | 0.433 | 0.319 | 0.299 | 0.310 | 0.326 | 0.405 | 0.32 | 0.34 | 0.04 |
| Gallbladder Wall | 1.33 | 1.26 | 1.14 | 1.13 | 0.875 | 0.803 | 0.840 | 0.987 | 0.986 | 0.963 | 0.99 | 1.03 | 0.17 |
| Left Colon | 0.594 | 0.558 | 0.632 | 0.593 | 0.808 | 0.553 | 0.521 | 0.565 | 0.582 | 0.778 | 0.59 | 0.62 | 0.09 |
| Small Intestine | 0.580 | 0.546 | 0.608 | 0.579 | 0.646 | 0.534 | 0.506 | 0.542 | 0.560 | 0.638 | 0.57 | 0.57 | 0.04 |
| Stomach Wall | 0.681 | 0.638 | 0.680 | 0.658 | 0.750 | 0.608 | 0.598 | 0.631 | 0.653 | 0.746 | 0.66 | 0.66 | 0.05 |
| Right Colon | 0.693 | 0.654 | 0.681 | 0.659 | 0.707 | 0.569 | 0.553 | 0.607 | 0.620 | 0.707 | 0.66 | 0.65 | 0.05 |
| Rectum | 0.489 | 0.459 | 0.536 | 0.503 | 0.630 | 0.480 | 0.447 | 0.471 | 0.490 | 0.594 | 0.49 | 0.51 | 0.06 |
| Heart Wall | 1.24 | 1.16 | 1.34 | 1.30 | 1.82 | 1.54 | 1.58 | 1.45 | 1.41 | 1.66 | 1.43 | 1.45 | 0.19 |
| Kidneys | 1.30 | 1.32 | 1.45 | 1.41 | 1.62 | 1.42 | 1.25 | 0.944 | 0.892 | 1.36 | 1.44 | 1.50 | 0.22 |
| Liver | 2.50 | 2.35 | 1.96 | 1.98 | 1.75 | 1.21 | 1.34 | 1.49 | 1.33 | 2.03 | 1.86 | 1.86 | 0.40 |
| Lungs | 0.592 | 0.556 | 0.591 | 0.571 | 0.707 | 0.526 | 0.518 | 0.543 | 0.552 | 0.696 | 0.56 | 0.59 | 0.06 |
| Ovaries |  |  |  |  | 0.645 |  |  |  |  | 0.612 | 0.63 | 0.63 | 0.02 |
| Pancreas | 0.778 | 0.731 | 0.767 | 0.739 | 0.904 | 0.655 | 0.643 | 0.695 | 0.710 | 0.959 | 0.74 | 0.76 | 0.10 |
| Prostate | 0.468 | 0.444 | 0.491 | 0.481 | - | 0.454 | 0.429 | 0.449 | 0.473 |  | 0.46 | 0.46 | 0.02 |
| Salivary Glands | 0.386 | 0.366 | 0.411 | 0.400 | 0.469 | 0.382 | 0.360 | 0.373 | 0.393 | 0.440 | 0.39 | 0.40 | 0.03 |
| Red Marrow | 0.760 | 0.662 | 1.01 | 0.745 | 0.916 | 0.729 | 0.631 | 0.713 | 0.673 | 0.875 | 0.74 | 0.77 | 0.12 |
| Osteogenic Cells | 0.620 | 0.555 | 0.772 | 0.621 | 0.720 | 0.603 | 0.536 | 0.589 | 0.579 | 0.684 | 0.61 | 0.63 | 0.07 |
| Spleen | 1.14 | 0.891 | 1.24 | 0.911 | 1.35 | 0.664 | 0.888 | 0.565 | 0.674 | 1.38 | 1.03 | 1.03 | 0.26 |
| Testes | 0.350 | 0.333 | 0.366 | 0.364 | - | 0.347 | 0.328 | 0.339 | 0.360 |  | 0.36 | 0.43 | 0.16 |
| Thymus | 0.594 | 0.560 | 0.632 | 0.612 | 0.758 | 0.647 | 0.641 | 0.625 | 0.630 | 0.713 | 0.62 | 0.59 | 0.05 |
| Thyroid | 0.428 | 0.405 | 0.448 | 0.437 | 0.512 | 0.417 | 0.398 | 0.411 | 0.430 | 0.484 | 0.43 | 0.43 | 0.02 |
| Urinary Bladder Wall | 0.452 | 0.429 | 0.477 | 0.467 | 0.465 | 0.444 | 0.418 | 0.435 | 0.459 | 0.439 | 0.46 | 0.48 | 0.07 |
| Uterus |  |  |  |  | 0.621 |  |  |  |  | 0.588 | 0.60 | 0.60 | 0.02 |
| Effective dose in total Body ICRP-103 (mSv/MBq) | **0.584** | **0.542** | **0.600** | **0.552** | **0.700** | **0.491** | **0.473** | **0.521** | **0.525** | **0.706** | **0.542** | **0.568** | **0.085** |

**Supplementary table 3.**
Individual absorbed dose to organs calculated using OLINDA/EXM 1.1

|  | Absorbed dose (mGy/MBq) | | | | | | | | | | | | |
| --- | --- | --- | --- | --- | --- | --- | --- | --- | --- | --- | --- | --- | --- |
| Patient | 1 | 2 | 3 | 4 | 5 | 6 | 7 | 8 | 9 | 10 | Median | Mean | SD |
| Target organs |  |  |  |  |  |  |  |  |  |  |  |  |  |
| Adrenals | 0.686 | 0.671 | 0.671 | 0.640 | 0.639 | 0.589 | 0.577 | 0.629 | 0.646 | 0.647 | 0.643 | 0.640 | 0.035 |
| Brain | 0.226 | 0.235 | 0.239 | 0.237 | 0.284 | 0.264 | 0.252 | 0.240 | 0.268 | 0.243 | 0.242 | 0.249 | 0.018 |
| Breasts | 0.285 | 0.289 | 0.286 | 0.287 | 0.326 | 0.307 | 0.302 | 0.292 | 0.314 | 0.293 | 0.293 | 0.298 | 0.014 |
| Galbladder Wall | 0.877 | 0.857 | 0.757 | 0.764 | 0.686 | 0.608 | 0.627 | 0.700 | 0.721 | 0.731 | 0.726 | 0.733 | 0.087 |
| Lower large intestine wall | 0.356 | 0.365 | 0.384 | 0.369 | 0.434 | 0.404 | 0.383 | 0.373 | 0.409 | 0.377 | 0.380 | 0.385 | 0.024 |
| Small intestine wall | 0.431 | 0.438 | 0.441 | 0.430 | 0.480 | 0.444 | 0.427 | 0.430 | 0.464 | 0.438 | 0.438 | 0.442 | 0.017 |
| Stomach wall | 0.439 | 0.441 | 0.431 | 0.428 | 0.478 | 0.434 | 0.424 | 0.431 | 0.471 | 0.445 | 0.437 | 0.442 | 0.018 |
| Upper large intestine wall | 0.464 | 0.468 | 0.455 | 0.451 | 0.485 | 0.445 | 0.433 | 0.442 | 0.475 | 0.453 | 0.454 | 0.457 | 0.016 |
| Heart wall | 0.986 | 0.950 | 1.08 | 1.06 | 1.26 | 1.33 | 1.37 | 1.22 | 1.21 | 1.13 | 1.17 | 1.16 | 0.142 |
| Kidneys | 1.14 | 1.19 | 1.30 | 1.27 | 1.32 | 1.33 | 1.18 | 1.57 | 1.44 | 1.66 | 1.31 | 1.34 | 0.170 |
| Liver | 2.25 | 2.13 | 1.75 | 1.77 | 1.28 | 1.07 | 1.20 | 1.49 | 1.46 | 1.59 | 1.54 | 1.60 | 0.384 |
| Lungs | 0.437 | 0.434 | 0.432 | 0.425 | 0.461 | 0.433 | 0.430 | 0.427 | 0.451 | 0.429 | 0.433 | 0.436 | 0.011 |
| Muscle | 0.323 | 0.330 | 0.326 | 0.325 | 0.369 | 0.342 | 0.331 | 0.327 | 0.356 | 0.332 | 0.331 | 0.336 | 0.015 |
| Ovaries | 0.383 | 0.391 | 0.407 | 0.392 | 0.455 | 0.423 | 0.403 | 0.395 | 0.431 | 0.399 | 0.401 | 0.408 | 0.022 |
| Pancreas | 0.633 | 0.620 | 0.611 | 0.592 | 0.618 | 0.554 | 0.545 | 0.583 | 0.623 | 0.608 | 0.610 | 0.599 | 0.030 |
| Red marrow | 0.637 | 0.573 | 0.869 | 0.627 | 0.663 | 0.660 | 0.572 | 0.626 | 0.599 | 0.610 | 0.627 | 0.644 | 0.085 |
| Osteogenic cells | 0.572 | 0.549 | 0.698 | 0.577 | 0.644 | 0.620 | 0.564 | 0.579 | 0.595 | 0.574 | 0.578 | 0.597 | 0.045 |
| Skin | 0.222 | 0.229 | 0.222 | 0.225 | 0.258 | 0.238 | 0.232 | 0.226 | 0.249 | 0.230 | 0.230 | 0.233 | 0.012 |
| Spleen | 0.924 | 0.728 | 0.998 | 0.727 | 0.950 | 0.554 | 0.503 | 0.722 | 1.02 | 0.948 | 0.826 | 0.807 | 0.186 |
| Testes | 0.244 | 0.259 | 0.244 | 0.257 | 0.312 | 0.287 | 0.278 | 0.261 | 0.296 | 0.266 | 0.264 | 0.270 | 0.022 |
| Thymus | 0.404 | 0.407 | 0.425 | 0.423 | 0.500 | 0.490 | 0.486 | 0.452 | 0.477 | 0.441 | 0.447 | 0.451 | 0.036 |
| Thyroid | 0.279 | 0.292 | 0.287 | 0.292 | 0.350 | 0.325 | 0.313 | 0.297 | 0.331 | 0.300 | 0.299 | 0.307 | 0.023 |
| Urinary bladder wall | 0.322 | 0.338 | 0.327 | 0.336 | 0.400 | 0.369 | 0.356 | 0.340 | 0.380 | 0.345 | 0.343 | 0.351 | 0.025 |
| Uterus | 0.367 | 0.380 | 0.380 | 0.379 | 0.444 | 0.411 | 0.394 | 0.382 | 0.422 | 0.388 | 0.385 | 0.395 | 0.024 |
| Effective dose in total Body ICRP-60 (mSv/MBq) | **0.493** | **0.483** | **0.506** | **0.470** | **0.507** | **0.472** | **0.440** | **0.480** | **0.482** | **0.490** | **0.483** | **0.482** | **0.019** |

**Supplementary Figure 1.**


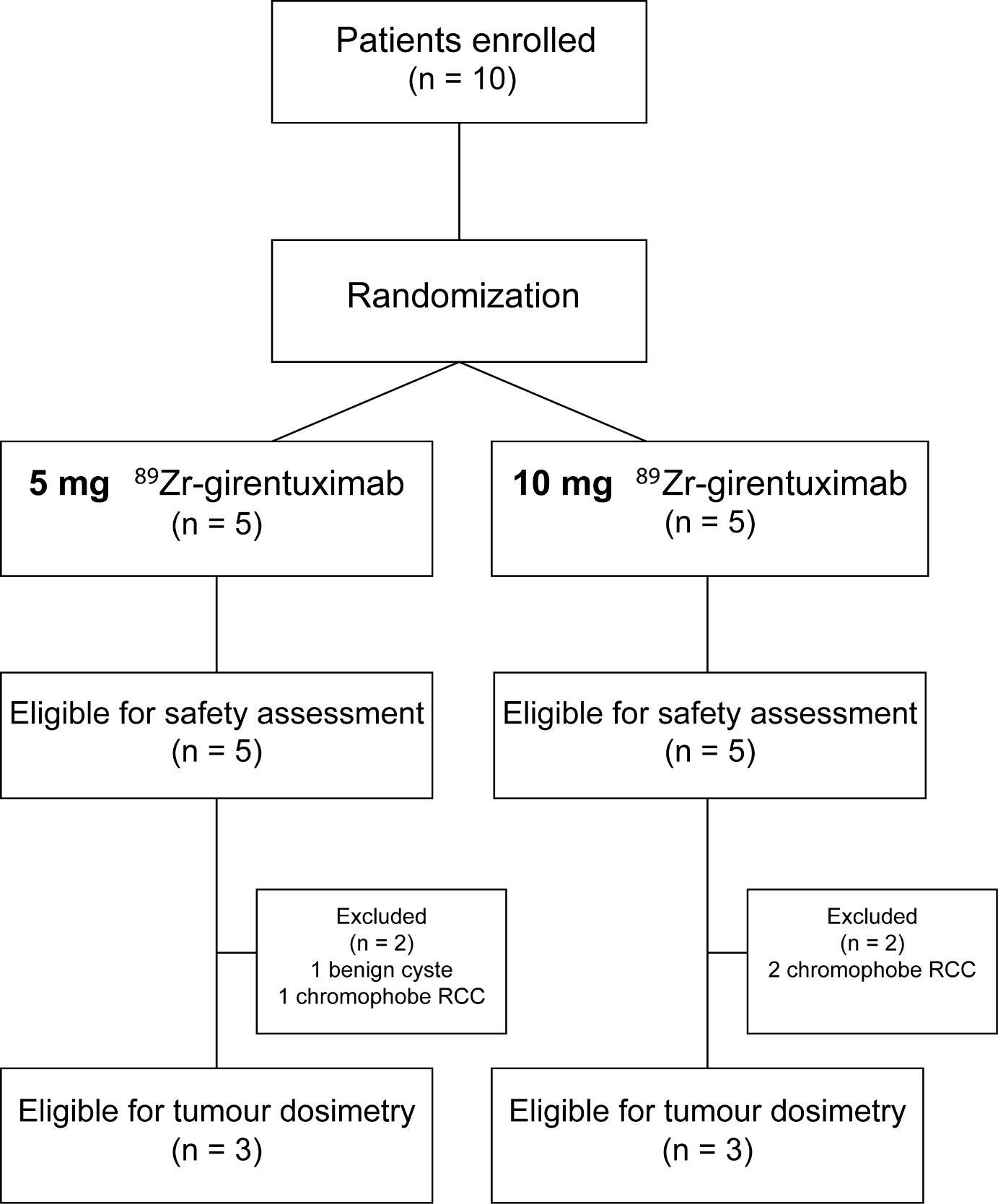


Supplemental Figure 1. Flow diagram of enrollment.
